# PATJ inhibits histone deacetylase 7 to control tight junction formation and cell polarity

**DOI:** 10.1007/s00018-023-04994-3

**Published:** 2023-10-25

**Authors:** Julia Fiedler, Thomas Moennig, Johanna H. Hinrichs, Annika Weber, Thomas Wagner, Tim Hemmer, Rita Schröter, Thomas Weide, Daniel Epting, Carsten Bergmann, Pavel Nedvetsky, Michael P. Krahn

**Affiliations:** 1 Department of Medical Cell Biology, Medical Clinic D, University Hospital of Münster, Albert-Schweitzer-Campus 1-A14, 48149 Münster, Germany; 2 Department of Medicine IV, Faculty of Medicine, Medical Center, University of Freiburg, 79106 Freiburg, Germany; 3 Medizinische Genetik Mainz, Limbach Genetics, 55128 Mainz, Germany

**Keywords:** Crumbs-complex, Tight junctions, HDAC7, Cell polarity

## Abstract

The conserved multiple PDZ-domain containing protein PATJ stabilizes the Crumbs-Pals1 complex to regulate apical-basal polarity and tight junction formation in epithelial cells. However, the molecular mechanism of PATJ’s function in these processes is still unclear. In this study, we demonstrate that knockout of PATJ in epithelial cells results in tight junction defects as well as in a disturbed apical-basal polarity and impaired lumen formation in three-dimensional cyst assays. Mechanistically, we found PATJ to associate with and inhibit histone deacetylase 7 (HDAC7). Inhibition or downregulation of HDAC7 restores polarity and lumen formation. Gene expression analysis of PATJ-deficient cells revealed an impaired expression of genes involved in cell junction assembly and membrane organization, which is rescued by the downregulation of HDAC7. Notably, the function of PATJ regulating HDAC7-dependent cilia formation does not depend on its canonical interaction partner, Pals1, indicating a new role of PATJ, which is distinct from its function in the Crumbs complex. By contrast, polarity and lumen phenotypes observed in Pals1- and PATJ-deficient epithelial cells can be rescued by inhibition of HDAC7, suggesting that the main function of this polarity complex in this process is to modulate the transcriptional profile of epithelial cells by inhibiting HDAC7.

**Supplementary Information:**

The online version contains supplementary material available at 10.1007/s00018-023-04994-3.

## Introduction

**Fig. 1:**
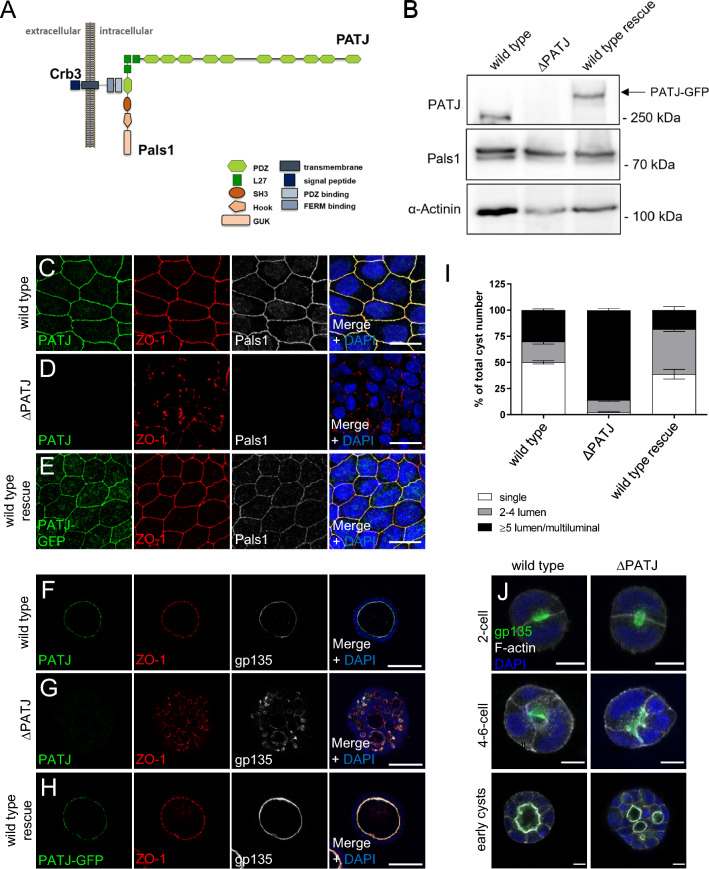
Knockout of PATJ results in TJ and polarity defects. **A** Scheme of the Crb complex in mammalian epithelial cells. In PATJ, point mutations found in CKD patients are indicated. **B** Western Blot analysis of MDCK cells with knockout of PATJ (MDCK∆PATJ) reveal a loss of protein expression. In wild-type rescue cells, PATJ-deficient MDCK cells were transduced with mouse PATJ-GFP (mPATJ-GFP). **C**–**E** Wild-type MDCK cells (**C**), PATJ-deficient cells (**D**) and MDCK∆PATJ cells with mPATJ-GFP-rescue were stained for the indicated antibodies. **F**–**H** Wild-type MDCK cells (**F**), PATJ-deficient MDCK cells (**G**) and rescue cells (**H**) were cultured in Matrigel and emerging cysts were stained with the indicated antibodies. **I** Quantification of cysts shown in **F**–**H**. Error bars are standard errors of the means. **J** Wild-type and PATJ-deficient MDCK cells were stained 24, 48 and 72 h after seeding into Matrigel for the indicated antibodies. Scale bars are 20 µm in **C**–**E**, 50 µm in **F**–**H** and 10 µm in **J**. See also Figs. S1 and S2

**Fig. 2:**
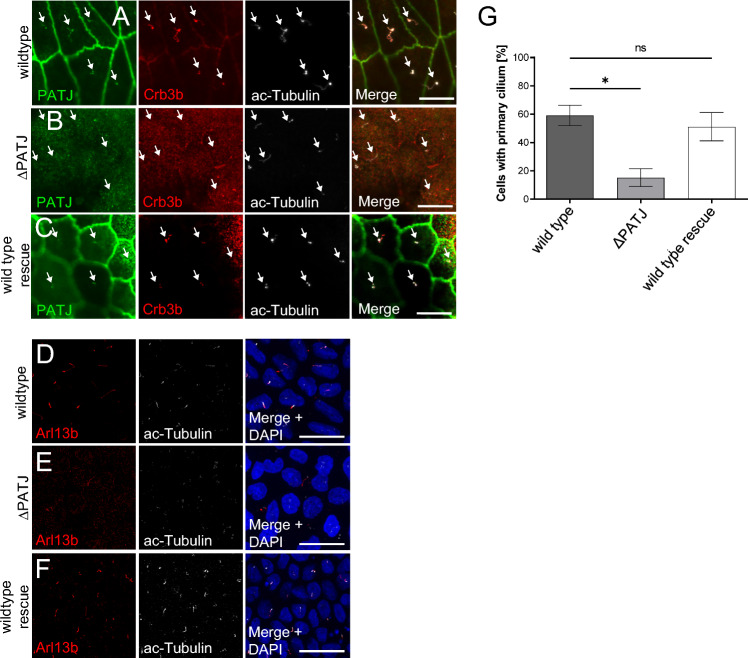
PATJ localizes to primary cilia and regulates cilia maintenance. **A**–**C** Immunostainings of PATJ, acetylated α-Tubulin (Ac-Tubulin) and Crb3b in wild-type MDCK cells (**A**), PATJ-deficient cells (**B**) and MDCK∆PATJ-cells with mPATJ-GFP rescue (**C**). Arrows indicate primary cilia. **D**–**F** Staining of primary cilia with Arl13b and ac-Tubulin demonstrates a reduction in primary cilia. **G** Quantification of primary cilia in the indicated cell lines. Error bars are standard errors of the means. Significance was determined by one-way ANOVA test and Bonferroni correction: *p < 0.05, ns not significant. Scale bars are 10 µm in **A**–**C** and 20 µm in **D**–**F**

The establishment of apical-basal polarity and tight junctions (TJ) is a crucial prerequisite for the function of epithelial cells, ensuring e.g. correct protein transport to either the apical or the basolateral plasma membrane as well as barrier formation and tissue homeostasis [1]. Apical-basal polarity is established by the balanced activity of highly conserved apical and basal polarity regulators. While the Scribble/Lethal(2) giant larvae/Discs large complex together with the kinase LKB1 and Par1 determine the basolateral plasma membrane domain, apical regulators include the PAR/aPKC complex as well as the Crumbs complex. The latter one consists of the transmembrane protein Crumbs (Crb, Crb3 in most epithelial cells) and its adapter protein Pals1 (Protein associated with Lin-7 one), which in turn recruits the multiple PDZ-domain containing protein PATJ (Pals1-associated tight junction protein) and Lin-7, another adapter protein, to the complex (Fig. 1A), [2]. Knockdown of any of these proteins in cultured mammalian epithelial cells results in the disassembly of the Crb-complex, disturbed apical-basal polarity, and TJ defects [3–6]. In vivo, *Crb3* knockout mice are viable but display defects in epithelial tissues of the lung and intestine as well as kidney cysts [7, 8]. Notably, the deletion of only one copy of Pals1 during kidney development results in kidney cysts [9]. For PATJ, in vivo data from *Drosophila* suggest a role in regulating the activation of non-muscle-myosin and the actin-regulator moesin [10–15], whereas apical-basal polarity is not affected in tissues lacking PATJ.

Apart from its role in TJ formation and apical-basal polarity, Crb and Pals1 have emerged as key regulators of several intracellular signaling cascades, including Hippo-, TGFβ- and mTOR signaling [9, 16–23]. Furthermore, we recently found that Pals1 regulates Rac1-dependent cell migration and metastasis of colorectal cancer cells by inhibiting Arf6, independently of its function within the Crb-complex [24].

Epithelial cells, as most quiescent mammalian cells, display one primary (immotile) cilium projecting from their apical plasma membrane. These organelles are supposed to function as mechano- and chemosensors, which initiate intracellular signaling cascades, thereby regulating various processes, including cell proliferation, migration, and -differentiation (reviewed by Ref. [25]). Consequently, loss or misfunction of primary cilia leads to severe defects in several organs, causing diseases summarized as ciliopathies [26, 27].

One disease associated with ciliopathies is polycystic kidney disease (PKD), which is characterized by multiple fluid-filled cysts within the kidney caused by increased proliferation of the cyst-lining epithelium and enhanced secretion of liquid (reviewed by Ref. [28]). PKD ultimately leads to loss of renal function and end-stage renal disease (ESRD). The most frequent form of PKD is the autosomal dominant PKD (ADPKD) with an incidence of 1:400–1000, accounting for around 5% of all patients suffering from ESRD (summarized by Ref. [29]). A wide range of mutations in the genes *PKD1* and *PKD2*, which encode for the proteins polycystin-1 (PC1) and polycystin-2 (PC2), have been identified in 85% or 15% of the patients, respectively. Both proteins are multi-pass transmembrane proteins, localized to primary cilia, and are supposed to function together as a mechanosensory unit transducing cilia-dependent signals. Focal cyst development and the relatively late onset of the disease are explained by the necessity of a “second hit”, a loss of heterozygosity or other somatic mutation in the normal *PKD* allele. Apart from *PKD1* and *PKD2*, which account for the vast majority of mutations associated with PKD, several other mutations associated with autosomal dominant or recessive PKD have been identified in cilia-associated and non-associated proteins, e.g. Fibrocystin, Nephrocystins or Bardet-Biedl-Syndrome Proteins [26].

Notably, knockout of Crb and heterozygous loss of Pals1 results in the formation of multiple cysts within the kidneys [7–9], raising the question, of whether the third component of the Crb-complex, PATJ is involved in the pathogenesis of PKD, too.

Therefore, in this study, we investigated the effects of PATJ knockout in kidney tubular epithelial cells (MDCK cells) and found a reduction in primary cilia in addition to defects in TJ assembly and lumen formation. On the molecular level, we revealed that PATJ binds to and inhibits histone deacetylase 7 (HDAC7), thereby regulating cell polarity and primary cilia formation. HDAC7 is a class IIa histone deacetylase, which assembles in the nucleus with transcriptional (co)repressors, thereby regulating several cellular processes such as cell proliferation, differentiation, and survival (for review see [30]).

## Materials and methods

### Yeast-two-hybrid assays

For yeast-two-hybrid assays, yeast cells (*Saccharomyces cerevisiae* strain Y190) were simultaneously transformed with the corresponding prey plasmid encoding full-length HDAC7 or C-terminus of HDAC7 (aa 826-937) fused to DNA-activation domain of GAL4 (in pDest22 vector) and the bait plasmid with mPATJ PDZ1-3, PDZ8-10 or Pals1 (negative control) fused to the DNA-binding domain of GAL4 (in pDest32), respectively. Interaction of bait and prey plasmids was tested by the growth of yeast cells on a selective medium containing 50 mM 3AT (3-amino 1,2,4,-triazole), but lacking leucine, tryptophane, and histidine (-L-T-H).

### Cell culture

MDCK-II cells (obtained from ATCC) were cultured in MEM (Sigma-Aldrich) medium supplemented with 5% FCS and 1% antibiotics (streptomycin/penicillin). All cell lines were cultivated at 37 °C under 5% CO_2_ atmosphere and passaged every 3–4 days. Transfections were performed with Lipofectamine™ 2000 (Thermo Fisher Scientific) or XtremeGene HP (SIGMA) according to the manufacturer’s instructions.

To establish stable knockout cell lines by using CRISPR/Cas9, cells were transfected with the following guides together with Cas9 from px459: PATJ#1: 5′- GAGACAGTCAAATTATTAGA-3′; PATJ#2: 5′-GATATAGAACGGCCTTCAAC-3′. Non-transfected cells were eliminated with 48 h puromycin selection and subsequently, single-cell clones were generated and analyzed for efficient knockout by western blot and sequencing. Stable mPATJ-GFP rescue cell lines were established by using the retroviral LT3GEPIR system. Knockdown of HDAC7 was achieved by the retroviral inducible pInducer10 system using the following shRNA targets: HDAC7 shRNA1: 5′-GCAGCGTGGTCAAGCAGAAGC-3′, HDAC7 shRNA2: 5′-shRNA targets: HDAC7 shRNA1: 5′-GCAGCGTGGTCAAGCAGAAGC-3′-3′. Expression was induced by the addition of 250 ng/ml doxycycline. The following inhibitors were used in this study: TMP195, 10 µM, Selleckchem #S8502), Tubastatin A, 10 µM, (Selleckchem #S0709), TC-H 106, 10 µM (Selleckchem #S6738), Trichostatin A, 500 nM (Santa Cruz Biotechnology #sc-3511), RO-3306, 10 µM (Santa Cruz Biotechnology #sc-358700). For induction of primary cilia, cells 7 days post-confluency were serum starved for 72 h before fixation and staining.

### 3D cyst formation assay

3D cell culture was performed in 8-well chamber slides (ibidi, #80826), pre-coated with 8 µl Cultrex Basement Membrane Extract Type 2 (Bio-Techne, #3533-005-02). Cells were trypsinized and a cell suspension at 20,000 cells/ml was prepared in a growth medium containing 2.5% Cultrex Basement Membrane Extract Type 2. 250 µl of the suspension was plated into a pre-coated 8-well chamber. Cells were grown for indicated times. The medium was removed every 48 h and replaced with fresh growth medium containing 2.5% Cultrex RGF Basement Membrane Extract. For immunofluorescence, cells were fixed with 4% PFA for 10 min at RT.

To measure the spindle orientation, cells were synchronized as described earlier [31]. Briefly, two days after seeding into the 3D culture, cells were treated with the cyclin-dependent kinase 1 inhibitor RO-3306 (10 µM) for 10 h, resulting in a cell cycle arrest at G_2_/M-phase transition. Subsequently, cells were released for 1 h in a medium without RO-3306.

### Immunofluorescence analysis and quantification of TJ

Cells were grown on coverslips and fixed with 4% PFA in phosphate buffer pH 7.4 for 10 min or with methanol at − 20 °C for 20 min. For immunofluorescence staining of ac-Tubulin, cells were incubated on ice for 30 min prior to fixation to reduce background signals. After washing three times with PBS cells were incubated for 1 h with PBS + 2.5% horse serum and 0.1% Triton X-100. Subsequently, primary antibodies were diluted in the same solution for 2 h at RT or overnight at 4 °C. After washing three times with PBST the coverslips were incubated with the secondary antibodies (diluted 1:500 in PBST + HS), DAPI (1:1000, Invitrogen Life Technologies) and Phalloidin (1:250, Santa Cruz #363796) for 1 h. Finally, coverslips were washed with PBS and mounted in Mowiol. The following primary antibodies were used: rabbit anti Arl13b (1:200, Proteintech #17711-1-AP), goat anti-Claudin7 (1:50, Santa Cruz #17670), rat anti-Crb3b (1:100, raised in this study), rat anti-E-Cadherin (1:100, Santa Cruz #59778), goat anti-GFP (1:500, Rockland #600101215), mouse anti-gp135 (1:50, DSHB #3F2/D8), mouse anti-HDAC7 (1:100, Santa Cruz #74563), mouse anti-Pals1 (1:100, Santa Cruz #365411), rabbit anti-Pals1 (1:100, Proteintech #17710-1AP), rabbit anti-PATJ (1:100, SIGMA #SAB2700561), mouse anti-Occludin (1:100, Santa Cruz #271842), mouse anti-acytelated-tubulin (1:1.000, SIGMA #T6793), rat anti-ZO1 (1:100, Santa Cruz #33725).

For the quantification of TJ defects, the intensity of ZO-1 staining was measured along the junction from one tricellular junction to the next using the freehand line tool in Fiji. To average results from multiple junctions, the fluorescence intensity was normalized to the highest value measured along each junction, and the length of the measured junction was set to a standardized length of 1. Intensities along the junctions were grouped into 20 bins. Results are presented as means ± SD from three independent experiments, with 30 junctions measured in each experiment.

### Cell lysates and western blot

Cell lysates were made with Laemmli buffer (western blot). SDS-PAGE and western blotting were performed according to standard procedures. The following primary antibodies were used: mouse anti-HDAC7 (1:500, Santa Cruz #74563), mouse anti-acytelated-tubulin (1:2.000, SIMGA #T6793), rabbit anti-α-Actinin (1:2.000, Cell Signaling #6487), mouse anti-Pals1 (1:200, Santa Cruz #365411), mouse anti-ß-Actin (1:1000, Santa Cruz #47778), rabbit anti-PATJ (1:100, SIGMA #SAB2700561).

### mRNA isolation and RNAseq

For RNAseq, cells were grown to postconfluence and RNA isolated using GenElute™ Mammalian Total RNA Miniprep Kit according to the manufacturer’s instructions.

Library preparation of the total RNA was performed with the NEBNext Ultra II RNA directional Kit (New England Biolabs) and single-read sequencing was performed using a NextSeq 500 System (Illumina) with a read length of 75 base pairs. Using a molecular barcode, the samples were demultiplexed (bcl2fastq2) to fastq data and quality controlled (FastQC). Trimmomatic was used for adapter trimming and read filtering. Reads were aligned to the reference genome GCA_014441545.1 using Hisat2. The aligned reads were sorted using samtools and counted into genes using htsec counts. The testing for differential expression was performed using the R package deseq2. The platform Generic Gene Ontology term mapper (University of Princeton) was used for GO term analysis.

### Statistical analysis

All data is presented as mean ± SEM of at least three independent experiments. Significance was determined by one one-way ANOVA test and Bonferroni correction using GraphPad Prism. The significance in Fig. 6 was determined by an unpaired *t*-test. ns > 0.05, *p < 0.05, **p < 0.01, ***p < 0.001.

## Results

### Knockout of PATJ results in TJ defects

Downregulation of PATJ by shRNA has already been described to disturb TJ formation in epithelial cells [4, 6]. Using CRISPR/Cas9-mediated gene editing, we generated a PATJ knockout in kidney tubular epithelial cells (Madin Darby Canine Kidney, MDCK∆PATJ cells, Fig. 1B and Fig. S1A). As a control for the specificity of MDCK∆PATJ-associated phenotypes, we investigated a second, independent knockout line (Fig. S1) and established a control line by re-expressing GFP-tagged murine PATJ (mPATJ-GFP) at close-to endogenous protein levels (Fig. 1B). In line with previous findings, knockout of PATJ results in severe defects in TJ formation reflected by a mislocalization of Zonula Occludens Protein-1 (ZO-1, Fig. 1C–E, quantified in Fig. S1C) and the TJ transmembrane proteins Occludin and Claudin-7, whereas the adherens junction marker E-Cadherin is not affected (Fig. S2A–F). When cultivated in the extracellular matrix, wild-type MDCK cells form hollow cysts with the apical plasma membrane directed towards the lumen and the basolateral domain facing outwards (Fig. 1F and I). In contrast, deletion of PATJ totally abolishes the capacity to form single-lumen cysts, but results in a multiple-lumen phenotype (Fig. 1G and I and Fig. S1E), which is rescued by re-expression of mPATJ-GFP (Fig. 1H, I and Fig. S1F).

After the first cell division in the extracellular matrix, MDCK cells form an apical membrane initiation site (AMIS) at the midbody between these two cells, which is a crucial step for the establishment of a single lumen [32]. Notably, MDCK∆PATJ cells correctly form the AMIS and the lumen during the first cell division, which is still maintained during the second division and only lost in later stages (Fig. 1J and Fig. S2G). During the growth of the cysts, the orientation of the division plane of each mitotic cell is essential to maintain a single lumen and defects in spindle alignments result in a multilayered epithelium. However, PATJ-deficient cells do not exhibit defects in the division plane in these early stages as estimated by quantification of mitotic spindle angles alignment (Fig. S2H). These data suggest that in three-dimensional cyst development, PATJ is essential for apical-basal polarization or directed protein transport in later stages but not for the initial polarization, the formation of the AMIS, and the spindle alignment during early cell divisions.

### PATJ controls the formation of primary cilia

A splice variant of Crb3 (Crb3b) has been shown to localize to primary cilia and to regulate cilia formation by association with importin-β [33]. To test, whether PATJ displays a similar localization and function, we stained endogenous PATJ in MDCK cells and found PATJ to colocalize with endogenous Crb3b and with the cilia markers acetylated tubulin (ac-Tub) and Arl13b (Fig. 2A and data not shown). Knockout of PATJ results in significantly less cilia upon serum starvation, which can be rescued by expression of mPATJ-GFP (Fig. 2D–G). Notably, the knockout of PATJ results in a displacement of Crb3b from the remaining primary cilia (Fig. 2B).

### PATJ interacts with HDAC7 to control cilia formation

**Fig. 3:**
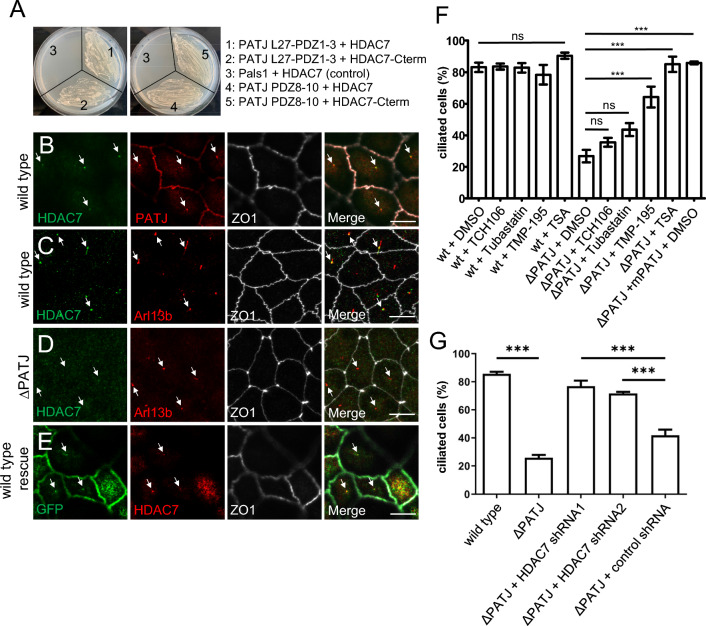
PATJ interacts with HDAC7 and inhibition of HDAC7 rescues cilia defects. **A** Yeast-two-hybrid experiments with full-length HDAC7 (#1,#3,#4) or C-terminus of HDAC7 (aa 826-937,#2 and #5) fused to DNA-binding domain together with mPATJ PDZ1-3 (1–2), PDZ8-10 (4–5) or Pals1 as negative control (3) fused to the DNA-activating domain. **B**–**E** Immunostainings of wild-type MDCK cells (**B**, **C**), PATJ-deficient cells (**D**) and MDCK∆PATJ cells with mPATJ-GFP rescue (**E**). **F** Quantification of the percentage of cells with primary cilia in wild-type MDCK cells and MDCK∆PATJ cells treated with indicated inhibitors or DMSO as control. **G** Quantification of cilia formation in the indicated cell lines. Error bars are standard errors of the means. Significance was determined by one-way ANOVA test and Bonferroni correction: ***p < 0.001, ns not significant. Scale bars are 10 µm. See also Fig. S3

To elucidate the molecular mechanism underlying cilia defects in PATJ-deficient cells, we investigated proteins associated with PATJ. In a genome-wide yeast-two-hybrid screen, HDAC7 has been identified as a potential interaction partner of PATJ [34]. Other HDACs have already been described to be involved in cilia disassembly or maintenance, in particular HDAC6, which deacetylates tubulin, thus destabilizing microtubules, leading to cilia disassembly [35]. Furthermore, HDAC3 and HDAC8 are essential during cilia formation [36], whereas HDAC2 promotes destruction of cilia [37]. HDAC7 contains a class II PDZ-binding motif at its C-terminus (Met-Asn-Leu). Using the yeast-two-hybrid system we verified that the multiple PDZ-domain containing protein PATJ directly interacts with the C-terminus of HDAC7 (Fig. 3A). Interestingly, several PDZ domains seem to be capable of binding HDAC7 as a construct of PDZ1-3 as well as PDZ8-10 interact with full-length HDAC7 or its C-terminus, whereas PDZ4-7 do not interact (Fig. 3A and data not shown). This is in line with previous data from Drosophila PATJ, demonstrating that several PDZ domains function in redundancy in vivo [11]. Furthermore, multiple PDZ domains of PATJ have already been described to interact with PC2 and TAZ [38]. In MDCK cells, endogenous HDAC7 can be detected at primary cilia, colocalizing with PATJ (in 67% of primary cilia, *n* > 100, *N* = 3) and Arl13b (Fig. 3B, C). Deletion of PATJ results in a displacement of HDAC7 from remaining cilia (only 12% of remaining cilia display staining for HDAC7, *n* > 100, *N* = 3, Fig. 3D), indicating that PATJ recruits HDAC7 to primary cilia.

To test, whether the modified activity of HDAC7 in the absence of PATJ is responsible for the cilia phenotypes in MDCK∆PATJ cells, we incubated these cells with compounds inhibiting all HDACs (Trichostatin, TSA), HDAC6 (Tubastatin), HDAC1, 2, 3 and 8 (TCH-106) or class IIa HDACs (HDAC4, 5, 7, 9; TMP195). In wild-type MDCK cells or MDCK∆PATJ cells with mPATJ-GFP rescue, inhibition of HDACs did not affect the number of cilia (Fig. 3F and data not shown), whereas in PATJ-deficient cells, inhibition of all HDACs (TSA) or inhibition of class IIa HDACs results in a significant increase in the number of primary cilia (Fig. 3F). In contrast, inhibition of HDAC1/3 or HDAC6 (TCH-106 and Tubastatin) did not substantially affect the number of cilia (Fig. 3F). To distinguish between HDAC7 and other class IIa HDACs more specifically, we generated an inducible knockdown of HDAC7 in MDCK∆PATJ cells using shRNA (Fig. S3A). Indeed, the downregulation of HDAC7 in PATJ-deficient cells restores cilia formation (Fig. 3G). Notably, tubulin acetylation, which is one critical mechanism in HDAC6-mediated cilia disassembly, is not affected in PATJ-deficient cells with or without HDAC7 inhibition (Fig. S3B), which argues for a different mechanism of HDAC7 regulating cilia formation/disassembly.

### Inhibition of HDAC7 restores polarity in PATJ-deficient cells

**Fig. 4:**
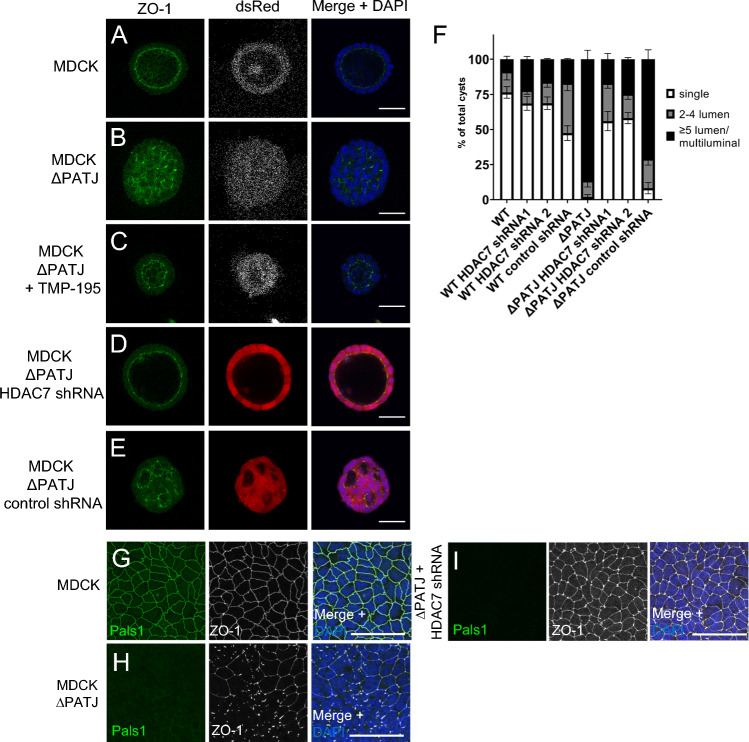
Inhibition of HDAC7 rescues polarity phenotypes of PATJ-deficient cells. **A**–**E** Immunostaining of wild-type MDCK cells (**A**), PATJ-deficient MDCK cells treated with DMSO (**B**) or TMP-195 (**C**) and MDCK∆PATJ cells transduced with shRNA against HDAC7 (**D**) or control shRNA (**E**), cultured in Matrigel with the indicated antibodies. **F** Quantification of lumen formation in the indicated cell lines. Error bars are standard error of the means. **G**–**I** Immunostaining of wild-type MDCK cells (**G**), PATJ-deficient MDCK cells (**H**) or MDCK∆PATJ cells transduced with shRNA against HDAC7 (**I**) with the indicated antibodies. Scale bars are 30 µm

Next, we investigated whether inhibition of HDAC7 can mitigate the polarity defects in MDCK∆PATJ cells. Incubation of these cells with TMP195 rescues the lumen defects observed in PATJ-depleted cells (Fig. 4A–C), restoring single lumen in the majority of cysts. Like chemical inhibition of HDAC7, shRNA-mediated downregulation of HDAC7 in PATJ-deficient cells results in a restored lumen formation (Fig. 4D–F). Notably, TMP195-treated cysts are much smaller than control cysts, implying the effects of this compound on cell proliferation, which is independent of HDAC7, as cells expressing shRNA directed against HDAC7 are not affected (Fig. 4A–F). Knockdown of HDAC7 in MDCK∆PATJ restores not only correct lumen formation but also rescues TJ defects (monitored by ZO-1 localization) in 3D (Fig. 4D, E) and 2D (Fig. 4G–I). These data indicate that inhibition of HDAC7 is the key function of PATJ by which it regulates correct lumen formation in three-dimensional space and cell polarity/TJ assembly as well as formation/maintenance of primary cilia.

### HDAC7 changes the transcriptional profile in PATJ-deficient cells

**Fig. 5:**
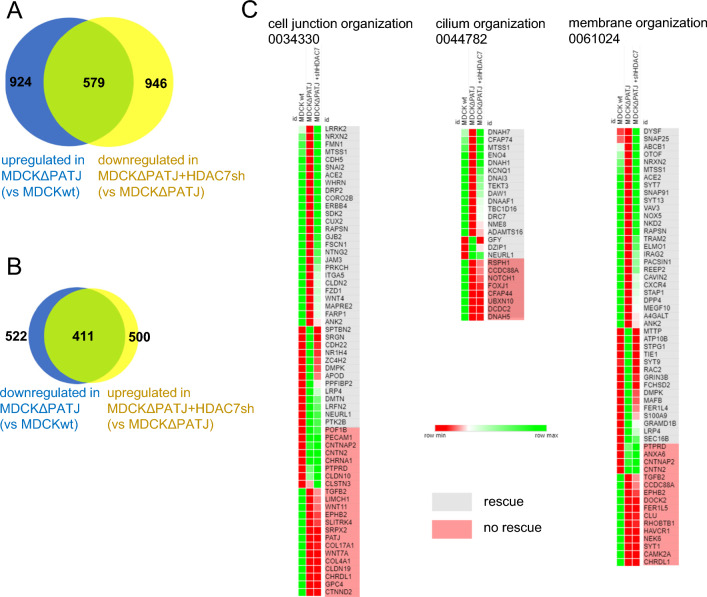
RNAseq analysis reveals HDAC7-dependent changes in the transcriptional profile. **A**, **B** Number of genes whose expressions were significantly (log2 fold change > 2) down-(**A**) or upregulated (**B**) in MDCK∆PATJ cells compared to wild-type MDCK cells (blue circle). Genes, which are up- (**A**) or downregulated (**B**) in MDCK∆PATJ + HDAC7-shRNA in comparison to MDCK∆PATJ are represented by yellow circle. Green represents genes, which are rescued in MDCK∆PATJ + HDAC7 shRNA cells. Sufficient rescue was assumed, if at least 25% of mRNA expression compared to wild type MDCK is restored. **C** Heat map of differentially regulated genes which are summarized in the indicated GO terms. Raw expression data are depicted according to the color code for wild-type MDCK (left row), MDCK∆PATJ (middle row) and MDCK∆PATJ + HDAC7 shRNA (right row). See also Fig. S4 for additional data

HDAC7 has been described to deacetylate Histones 3 and 4, thereby functioning as a transcriptional co-regulator [reviewed by 30]. We therefore analyzed the gene expression pattern of MDCK∆PATJ cells in comparison to control cells by RNA-sequencing (RNAseq). Indeed, we found 924 genes to be significantly upregulated and 522 genes to be significantly downregulated in PATJ-deficient cells (Fig. 5A and B). Notably, the majority of downregulated/upregulated genes were rescued upon shRNA-mediated downregulation of HDAC7 (579 and 411, respectively). GO-term analyses revealed an enrichment of genes involved in the organization of cell junctions, cilia, and membranes (Fig. 5C) as well as extracellular matrix and cytoskeleton organization (Fig. S4). Like the overall gene expression changes, most genes in the selected GO terms, which are up- or down-regulated in PATJ-deficient cells, were rescued in HDAC7-shRNA expressing cells: 26/40 of downregulated and 13/21 of upregulated genes in cell junction organization, 14/22 of downregulated and 3/3 of upregulated genes in cilium organization and 25/38 of downregulated and 15/19 of upregulated genes in membrane organization (Fig. 5C).

These data suggest that deletion of PATJ in epithelial cells results in substantial changes in the transcriptional profile of these cells, which are due to an aberrant activation of HDAC7. Many differentially expressed genes are involved in processes, which are closely related to the phenotypes observed in PATJ-deficient cells, in particular cell junction assembly, membrane organization, primary cilia formation, cytoskeleton, and organization of extracellular matrix.

### PATJ inhibits HDAC7 independently of its canonical role within the Crb-complex

**Fig. 6:**
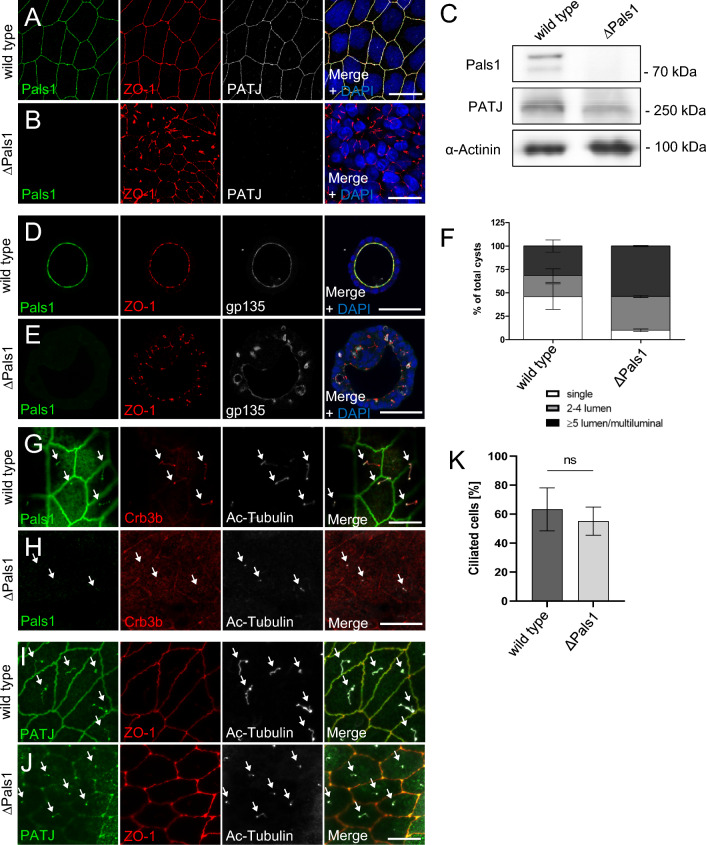
Loss of Pals1 results in TJ and lumen formation defects but does not affect cilia formation. **A**, **B** Immunostaining of wild-type MDCK cells (**A**) and Pals1-deficient MDCK cells (**B**) with the indicated antibodies. **C** Western blot analysis of wild-type MDCK and MDCK∆Pals1 cells. **D**, **E** Immunostaining of wild-type MDCK (**D**) and MDCK∆Pals1 (**E**) cells cultured in Matrigel with the indicated antibodies. **F** Quantification of lumen formation in the indicated cell lines. Error bars are standard errors of the means. **G** Pals1 colocalizes with Crb3b at primary cilia, which are labelled with ac-Tubulin. **H** Immunostaining of MDCK∆Pals1 cells with Pals1, Crb3b and ac-Tubulin. **I**, **J** PATJ is mostly lost from TJ in Pals1-depleted cells (**J**) but still localizes to primary cilia (arrows). **L** Quantification of primary cilia number in wild-type and Pals1-deficient MDCK cells reveals no significant differences. Error bars are standard errors of the means. Scales bars are 20 µm in **A**–**B** and **G**–**J** and 50 µm in **D**–**E**

PATJ has been described as an essential component of the Crb-complex, heterodimerizing with Pals1 via their L27-domains and thereby stabilizing Crb-Pals1 at the apical junctional region [39]. Additionally to PATJ, the Crb3 splice variant Crb3b as well as the canonical interaction partner of PATJ, Pals1 are localized to primary cilia (Fig. 6G) [33, 40]. To test, whether Pals1 is involved in PATJ/HDAC7-mediated control of ciliogenesis, we established a Pals1-knockout cell line (Fig. 6A–C). These cells display TJ and lumen formation defects similar to MDCK∆PATJ cells, albeit less severe as in 2D, ZO-1 is disturbed but still often localizes to cell–cell junctions and in 3D some single lumen cysts and more cysts with 2–4 lm can be detected (Fig. 6A–F). This is in line with previous reports using shRNA-mediated downregulation of Pals1, which demonstrated that TJ formation is delayed but not entirely disrupted [5, 9]. In Pals1-deficient cells, PATJ is displaced from cell–cell contacts, too, whereas its overall expression is only slightly affected (Fig. 6A–C). In contrast, PATJ is not lost from primary cilia, whereas Crb3b staining is reduced (Fig. 6G–J). Consequently, knockout of Pals1 did not affect the number of primary cilia (Fig. 6K), suggesting that Pals1 is not essential for PATJ regulating primary cilia formation by inhibiting HDAC7.

**Fig. 7:**
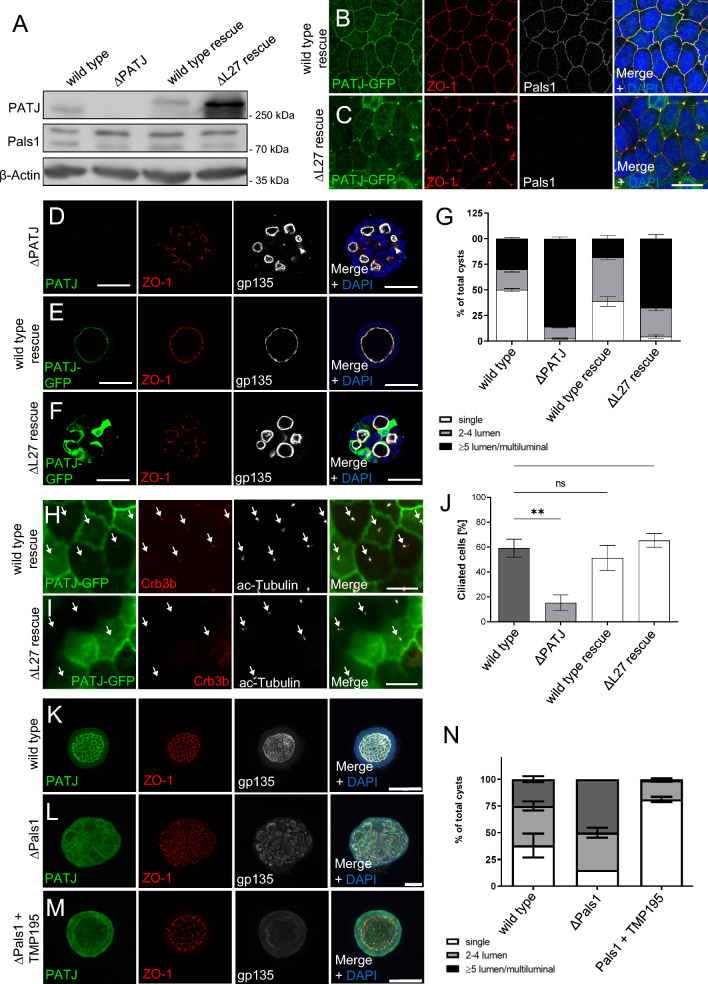
Pals1-binding deficient PATJ mimics polarity but not cilia defects. **A** Western blot of wild type MDCK, MDCK∆PATJ and MDCK∆PATJ transduced with wild type mPATJ (wild-type rescue) or mPATJ∆L27. **B**, **C** Immunostainings of the indicated cell lines with ZO-1 and Pals1 reveal TJ defects and a mislocalization of Pals1 from the TJ in case of PATJ-deficient and PATJ∆L27 expressing cells, whereas wild-type PATJ rescues TJ defects. **D**–**F** Indicated cell lines were cultured in Matrigel and stained with the indicated antibodies. **G** Quantification of lumen formation in cysts of the indicated cell lines. Error bars are standard error of the means. **H**, **I** Immunostaining of PATJ-GFP variants demonstrates a failure of PATJ∆L27 to localize to primary cilia. **J** Quantification of primary cilia formation upon serum starvation. Error bars are standard error of the means. **K**–**N** Inhibition of HDAC7 by TMP195 rescues single lumen formation defects in Pals1 deficient MDCK cells but not junctional PATJ localization. Error bars are standard errors of the means. Significance was determined by one-way ANOVA test and Bonferroni correction: *p < 0.05, ns not significant. Scales bars are 20 µm in **B**–**D** and **H**, **I**, 30 µm in **D**–**F** and 50 µm in **K**–**M**

To further test whether the interaction of PATJ and Pals1 is essential for PATJ’s function, we established a PATJ-deficient rescue cell line expressing a variant of PATJ lacking the L27 domain (PATJ∆L27, Fig. 7A), thus being unable to bind to Pals1. In cells cultured in 2D, expression of PATJ∆L27 recapitulates the TJ defects observed in Pals1-deficient cells with disturbed ZO-1 signal which still accumulates at tricellular junctions and to some degree at bicellular junctions, too (Fig. 7B, C). Notably, PATJ∆L27 seems to be more stable than wild-type PATJ (Fig. 7A) and mutant protein predominately localizes to tricellular junctions (Fig. 7C). In three-dimensional cyst assays, Pals1-binding deficient PATJ was not able to fully restore single-lumen formation but exhibited a phenotype similar to the knockout of Pals1 (Fig. 7D–G).

Although PATJ∆L27 is not capable of localizing to primary cilia (Fig. 7H, I) and Crb3b is displaced from these cilia, the overall number of primary cilia is not affected in PATJ∆L27 rescue cells (Fig. 7J).

Of note, inhibition of HDAC7 in MDCK∆Pals1 cells restores TJ and the formation of single-lumen cysts but not junctional localization of PATJ (Fig. 7K–N). Vice versa, inhibition of HDAC7 in PATJ-deficient cells restored TJ assembly to a similar extent without targeting Pals1 to cell–cell junctions (Fig. 4G–I). These data suggest that inhibition of HDAC7 is the major function of PATJ/Pals1 in regulating TJ assembly and apical-basal polarity.

## Discussion

In this study, we show that deletion of PATJ in kidney tubular epithelial cells disturbs TJ assembly, abolishes apical-basal polarity, and decreases the number of primary cilia. PATJ directly binds to and inhibits HDAC7, which is responsible for the transcriptional reprogramming of PATJ-deficient cells. Our Y2H results demonstrate that PATJ directly interacts with HDAC7 and the fact that knockout of Pals1 exhibits similar TJ and polarity phenotypes as PATJ-knockout which can both be rescued by inhibition of HDAC7 suggests that the Crb apical polarity complex is involved in the regulation of HDAC7, thereby controlling apical-basal polarity and TJ formation.

Different HDACs have already been described to be involved in the regulation of primary cilia: HDAC6 deacetylates α-tubulin, resulting in the destabilization of microtubules and subsequently cilia disassembly [41–44]. Controversially, inhibition of HDAC6 in a murine *Pkd1*-mutant PKD model reduces cysts growth, indicating that HDAC6 plays different roles in PKD, including regulating primary cilia formation, cell proliferation and cAMP-activated CFTR-channels responsible for liquid secretion [45–47]. In pancreatic ductal adenocarcinoma, HDAC2 enhances the disassembly of primary cilia, likely by promoting Aurora-A expression [37]. In contrast, HDAC3 and HDAC8 have been reported to positively regulate cilia formation and -morphology in retinal pigment epithelial and kidney proximal tubule cells, although the underlying mechanism is still unclear [36].

In *Pkd2*-deficient mice, the reduction of HDAC5 inhibits cyst formation by enhancing MEF2C-dependent transcription [48]. However, in contrast to HDAC6, HDAC5 is proposed to function downstream of primary cilia, transducing mechanical signals from cilia into transcriptional programs. Notably, in the nucleus murine HDAC7 binds to Nuclear receptor corepressor 2 (Ncor2) and assembles together with HDAC5 and the transcriptional corepressor Sin3a to form a multimeric gene repression complex [49]. In human cells, HDAC7 interacts with HDAC3 and NCOR1 in a similar way [50].

Our transcriptome analysis of PATJ-deficient cells with and without inhibition of HDAC7 reveals enrichment of differentially expressed genes, which are implicated in cilia organization. Strikingly, several genes, which are downregulated in PATJ-deficient cells and rescued upon downregulation of HDAC7, have already been described to be essential for the formation of cilia, e.g. DCDC2, MTSS1, Girdin (CCDC88A), Dynein Heavy Chain 5, DZIP1, NME8, RSPH1 and KCNH1 [51–58].

Thus, we conclude that PATJ functions to inhibit HDAC7 to prevent the repression of genes which regulate the assembly of primary cilia. Of note, this new function of PATJ is independent of its canonical interaction partner Pals1 because a PATJ variant, which does not bind to Pals1 is still capable of rescuing the cilia defects. Albeit both proteins colocalize at primary cilia, Pals1-binding-deficient PATJ is not targeted to cilia but still rescues cilia-phenotypes in PATJ-deficient cells, suggesting that inhibition of HDAC7 by PATJ is not restricted to primary cilia targeting of the proteins. Moreover, Crb3b localization at primary cilia is not fully rescued by PATJ∆L27 and Crb-mutants in zebrafish display shortened but not less cilia [59]. Finally, Crb3b is displaced from primary cilia in Pals1-deficient cells, although the overall number of cilia is not affected, all arguing against the role of Crb3b in the mechanism of PATJ regulating cilia formation.

Surprisingly, inhibition or downregulation of HDAC7 in PATJ-deficient cells does not only rescue cilia formation but also TJ- and apical-basal polarity defects. Our RNAseq data demonstrate the downregulation of numerous genes involved in cell adhesion, cell junction formation, membrane organization, extracellular matrix, vesicle transport, and regulation of the cytoskeleton, all processes which are essential for the establishment of the TJ and apical-basal polarity. Of note, in contrast to other regulators of apical-basal polarity and TJ, such as Cingulin [60], PATJ-deficient cells establish the AMIS and a single lumen between two and four dividing cells correctly at first but fail to maintain this single lumen later on during cyst development. Like its role in ciliogenesis, the function of PATJ/HDAC7 regulating apical-basal polarity and TJ formation are likely to be independent of Pals1, as these processes are rescued upon HDAC7 inhibition without the recruitment of Pals1 to the apical junctions. Nonetheless, deletion of Pals1 in epithelial cells results in a similar polarity- (but not cilia-) phenotype as knockout of PATJ, which is rescued by inhibition of HDAC7. Thus, the main function of PATJ/Pals1 in the context of apical-basal polarity and TJ regulation is the inhibition of HDAC7 to control the reprogramming of epithelial cells into differentiation. Further studies are necessary to investigate, which HDAC7-regulated pathways are essential in this context.

### Supplementary Information

Below is the link to the electronic supplementary material.

Supplementary file 1: Fold changes of RNAs isolated from MDCK∆PATJ cells versus wild type MDCK cells. Isolation and RNAseq was performed as described in Materials and Methods section. (XLSX 2056 KB)

Supplementary file 2: Fold changes of RNAs isolated from MDCK∆PATJ + HDAC7-shRNA cells versus MDCK∆PATJ cells. Isolation and RNAseq was performed as described in Materials and Methods section. (XLSX 2044 KB)

**Figure S1** Generation of MDCK∆PATJ cell lines. **A** Guide design and sequencing results from MDCK∆PATJ #1 (upper panel) and MDCK∆PATJ #2 (lower panel). Both cell lines exhibit a single base pair insertion, resulting in a shift of the open reading frame. **B** Western blot of MDCK∆PATJ#2 of PATJ-GFP variants demonstrates the absence of endogenous PATJ. **C** Quantification of TJ defects in wild type and PATJ-deficient cells. ZO-1 staining was used as described in the methods section. *N* = 3 with *n* = 30. Error bars represent standard deviation. **D**–**F** MDCK wild type (**C**), MDCK∆PATJ#2 (**D**) and MDCK∆PATJ#2 + mPATJ-GFP (**E**) cells were cultured in Matrigel and stained with the indicated antibodies (PDF 501 KB)

**Figure S2** PATJ-deficient MDCK cells display disturbed TJ but no defects in early cyst development. **A**–**F** Immunostaining of E-Cadherin and Pals1 in MDCK wild type (**A** and **D**), MDCK∆PATJ (**B** and **E**) and MDCK∆PATJ + mPATJ-GFP (**C** and **F**) reveals no changes in junctional E-Cadherin localization, whereas Pals1 is totally displaced from the cell–cell contacts and staining of Claudin-7 and Occludin are disturbed in PATJ-deficient cells. **G** Quantification of single lumen in early cytogenesis in wild type and PATJ-deficient cells. **H** Quantification of the division angle and immunostainings of the mitotic spindle in wild type and MDCK∆PATJ cells stained with. Scale bars 20 µm in A-F and 10 µm in **H** (PDF 755 KB)

**Figure S3** Downregulation of HDAC7 does not affect Tubulin acetylation. **A** Western blot of wild type MDCK cells, MDCK∆PATJ cells and MDCK∆PATJ cells expressing different HDAC7 shRNAs or a control shRNA demonstrate a downregulation of HDAC7 in all three shRNAs tested. **B** Western blot of the indicated cell lines with ac-Tubulin reveals no differences in Tubulin acetylation (PDF 209 KB)

**Figure S4** Transcriptome analysis of PATJ-deficient cells reveals changes in the expression of genes involved in various cellular processes. Heat maps of genes, which are significant (at least fourfold) down- or upregulated in PATJ-deficient cells compared to wild type MDCK cells, grouped in the indicated GO-terms. A rescue was assumed if at least 25% of expression is restored upon downregulation of HDAC7 in PATJ-deficient cells (PDF 585 KB)

## Data Availability

All used materials are commercially available. All data supporting the findings described in this study are available from the corresponding author upon reasonable request.
