# Supplementary figures and images for: PATJ inhibits histone deacetylase 7 to control tight junction formation and cell polarity
Source: Cell Mol Life Sci. 2023 Oct 25;80(11):333. doi: 10.1007/s00018-023-04994-3 (PMC10600057; doi:10.1007/s00018-023-04994-3)

Figure S1

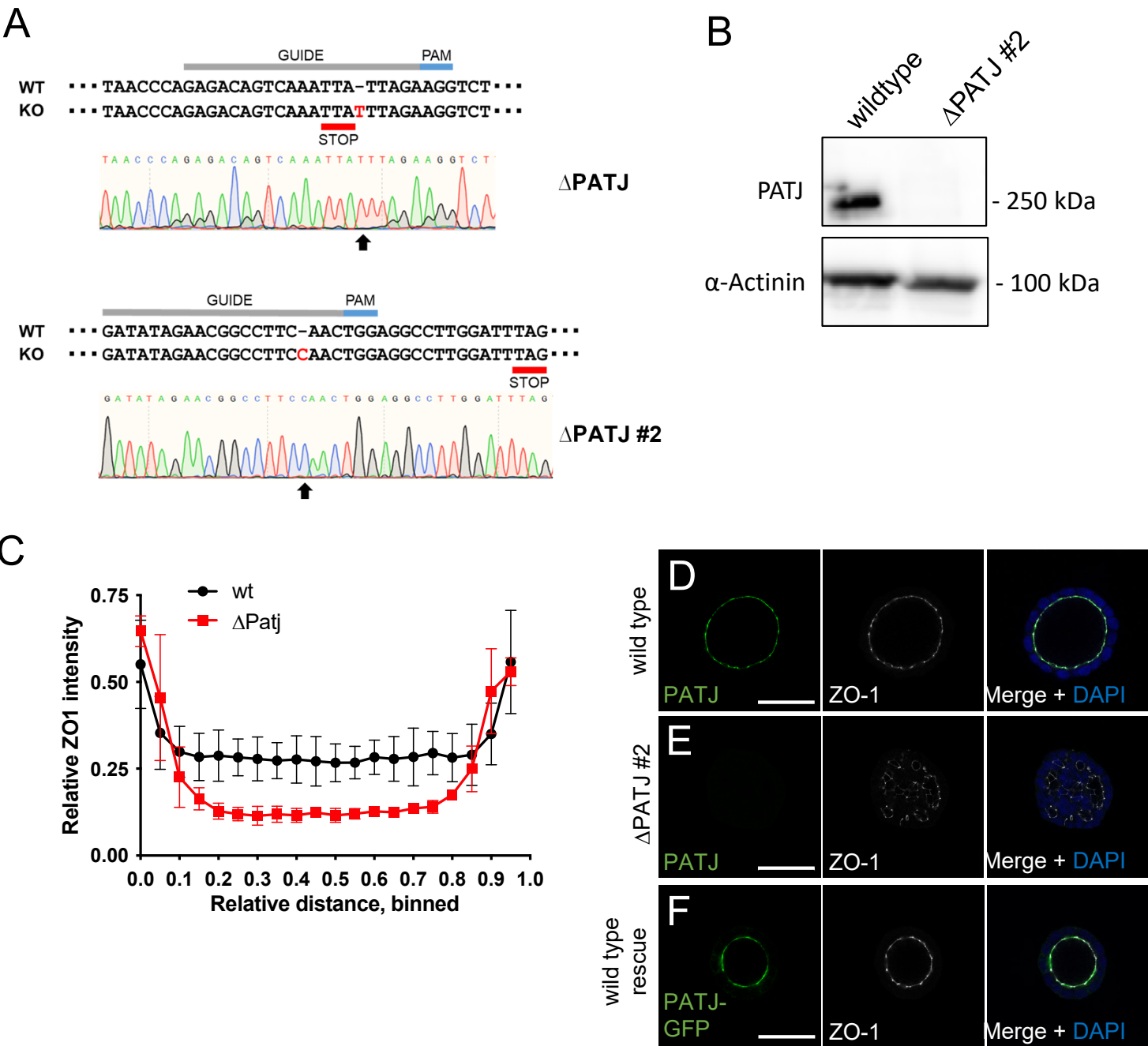

Supplement: Supplementary file 3 — Figure S1 Generation of MDCK∆PATJ cell lines. A Guide design and sequencing results from MDCK∆PATJ #1 (upper panel) and MDCK∆PATJ #2 (lower panel). Both cell lines exhibit a single base pair insertion, resulting in a shift of the open reading frame. B Western blot of MDCK∆PATJ#2 of PATJ-GFP variants demonstrates the absence of endogenous PATJ. C Quantification of TJ defects in wild type and PATJ-deficient cells. ZO-1 staining was used as described in the methods section. N = 3 with n = 30. Error bars represent standard deviation. D–F MDCK wild type (C), MDCK∆PATJ#2 (D) and MDCK∆PATJ#2 + mPATJ-GFP (E) cells were cultured in Matrigel and stained with the indicated antibodies (PDF 501 KB) [file 18_2023_4994_MOESM3_ESM.pdf]

Figure S2

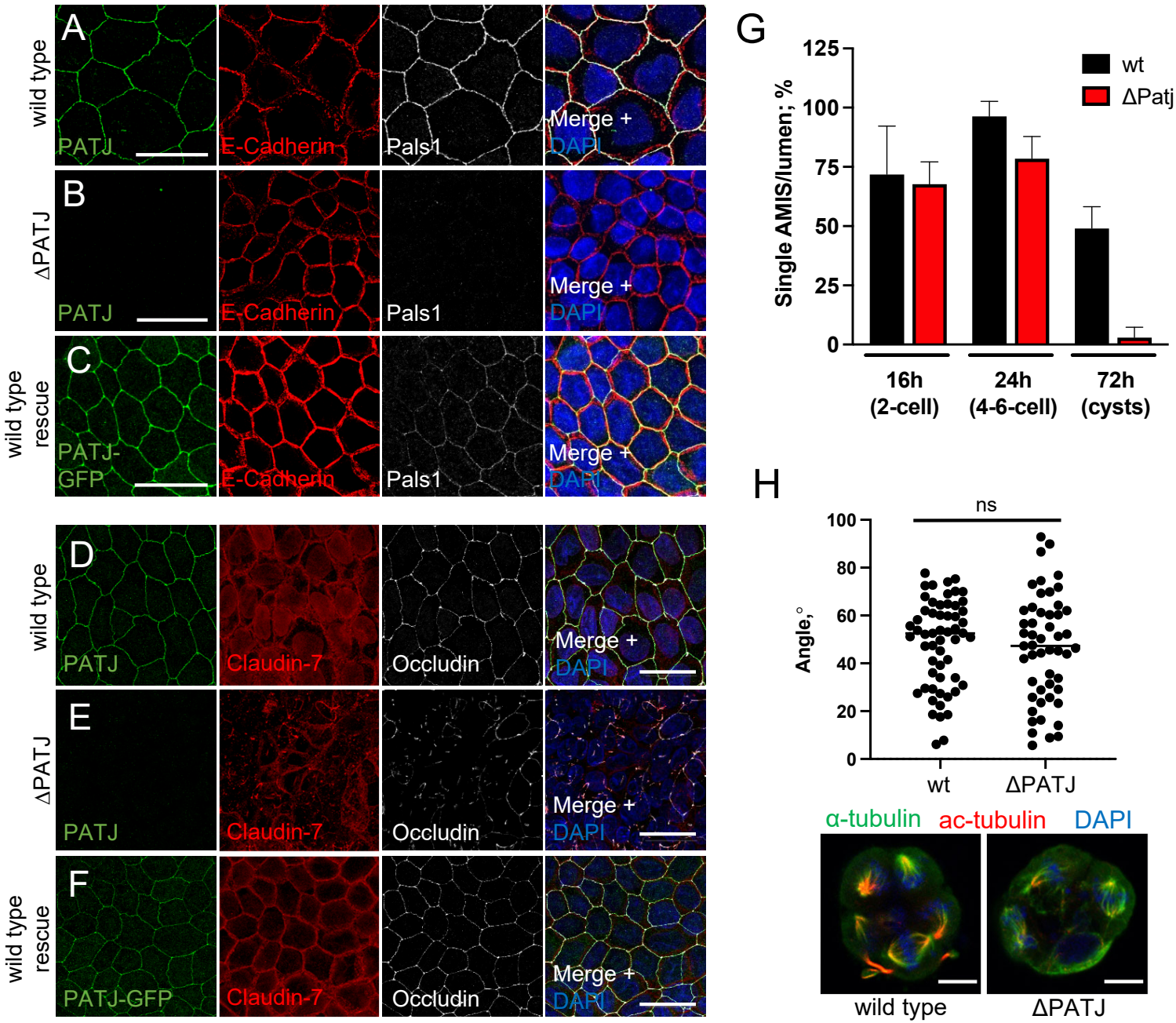

Supplement: Supplementary file 4 — Figure S2 PATJ-deficient MDCK cells display disturbed TJ but no defects in early cyst development. A–F Immunostaining of E-Cadherin and Pals1 in MDCK wild type (A and D), MDCK∆PATJ (B and E) and MDCK∆PATJ + mPATJ-GFP (C and F) reveals no changes in junctional E-Cadherin localization, whereas Pals1 is totally displaced from the cell–cell contacts and staining of Claudin-7 and Occludin are disturbed in PATJ-deficient cells. G Quantification of single lumen in early cytogenesis in wild type and PATJ-deficient cells. H Quantification of the division angle and immunostainings of the mitotic spindle in wild type and MDCK∆PATJ cells stained with. Scale bars 20 µm in A-F and 10 µm in H (PDF 755 KB) [file 18_2023_4994_MOESM4_ESM.pdf]

Supplementary Figure 3

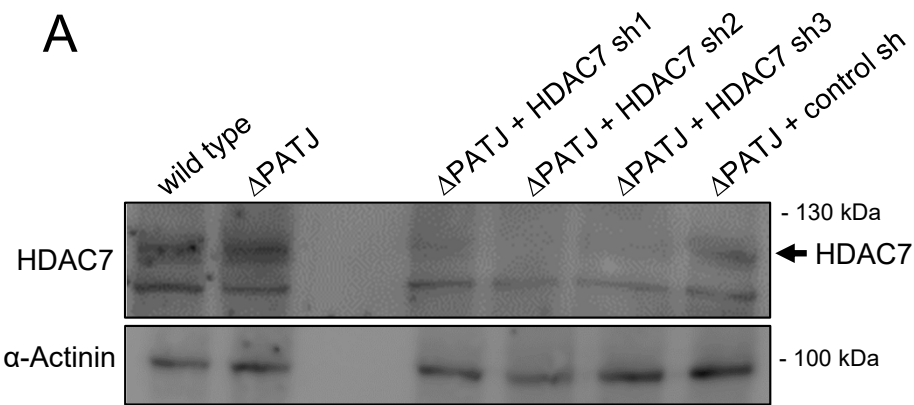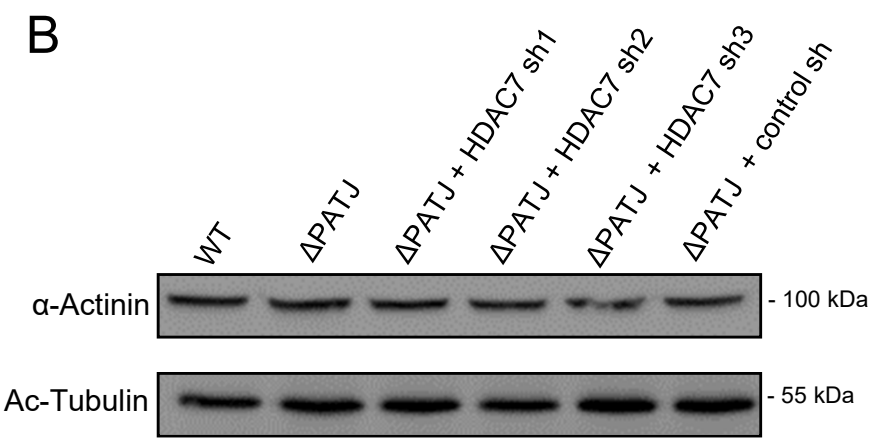

Supplement: Supplementary file 5 — Figure S3 Downregulation of HDAC7 does not affect Tubulin acetylation. A Western blot of wild type MDCK cells, MDCK∆PATJ cells and MDCK∆PATJ cells expressing different HDAC7 shRNAs or a control shRNA demonstrate a downregulation of HDAC7 in all three shRNAs tested. B Western blot of the indicated cell lines with ac-Tubulin reveals no differences in Tubulin acetylation (PDF 209 KB) [file 18_2023_4994_MOESM5_ESM.pdf]

# Figure S4

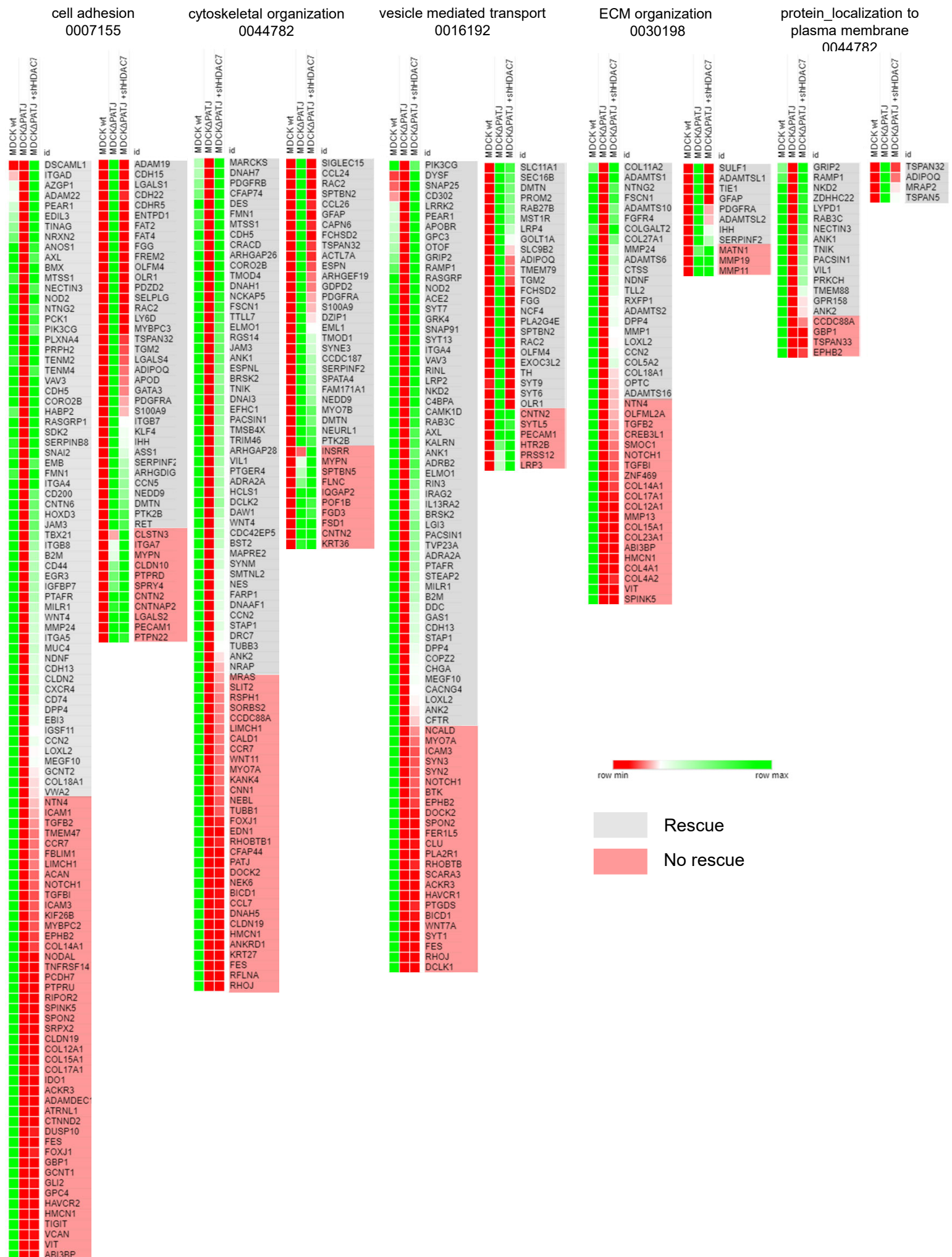

Supplement: Supplementary file 6 — Figure S4 Transcriptome analysis of PATJ-deficient cells reveals changes in the expression of genes involved in various cellular processes. Heat maps of genes, which are significant (at least fourfold) down- or upregulated in PATJ-deficient cells compared to wild type MDCK cells, grouped in the indicated GO-terms. A rescue was assumed if at least 25% of expression is restored upon downregulation of HDAC7 in PATJ-deficient cells (PDF 585 KB) [file 18_2023_4994_MOESM6_ESM.pdf]
